# Strengthening the Backbone: Government-Academic Data Collaborations for Crisis Response

**DOI:** 10.2196/64726

**Published:** 2024-11-28

**Authors:** Rick Yang, Alina Yang

**Affiliations:** 1 Harvard College, Cambridge, MA, United States; 2 Scarsdale High School, Scarsdale, NY, United States

**Keywords:** data infrastructure, data sharing, cross-sector collaboration, government-academic partnerships, public health, crisis response

We read with great interest the recent commentary detailing the need to bolster government-academic data infrastructures for more effective public health crisis response [1]. The authors present a compelling case for enhancing government-academic collaborations to improve data sharing during public health emergencies, advocating a shift from ad hoc collaborations to more robust and enduring partnerships. Nonetheless, certain points raised by the authors may benefit from a more thorough discussion and examination of the practical challenges and unrealized limitations inherent in such collaborations.

While the commentary underscores the critical role of data sharing in managing public health crises, the authors gloss over the substantial logistical and ethical challenges associated with cross-sector data sharing. For example, ensuring data privacy and security would present a significant hurdle. Societal experiences during the COVID-19 pandemic highlighted the complexities in maintaining the confidentiality of sensitive health data while also enabling timely access for research purposes [2]. Thus, a key step for successful government-academic collaboration will be the development of comprehensive data governance policies.

The authors’ vision for shifting from one-time data-sharing events to sustained partnerships must be tempered with a realistic understanding of the resources required to maintain such partnerships: ongoing funding, dedicated personnel, continuous training, among others [3]. Although there is indeed an underinvestment in public health data infrastructures, what are some possible actionable solutions to secure long-term funding, government grants, or private sector partnerships? The discussion would be greatly enriched if such strategies were outlined in greater detail.

It is also important to address the potential need for bidirectional learning and capacity building when considering ways to leverage academic expertise in data analysis. For instance, government agencies can offer invaluable contextual insights and access to extensive datasets, while academic researchers bring methodological rigor [4]. Continuous knowledge exchange and capacity building on both sides lay the foundation for effective collaborations. Such reciprocal learning provides public health response teams with enhanced quality of data analysis while ensuring that research findings are relevant and actionable for policymaking.

It is clear that government-academic collaborations can mitigate data quality issues and, in turn, enrich the data-sharing ecosystem. By further analyzing the methodological challenges of integrating data from disparate sources and their roots, such as variations in data collection methods, definitions, and standards, researchers and multi-stakeholder teams can develop standardized protocols for data collection and processing across different sectors. Additionally, investing in technologies that facilitate data harmonization and interoperability can supplement the effectiveness of these collaborations [5].

This commentary provides a timely framework for bolstering data sharing during public health crises in this day and age. Nonetheless, we propose a more nuanced analysis addressing issues related to data governance, resource allocation, bidirectional learning, and data standardization. Only by considering the complexities of implementing such cross-sector networks can we begin to brainstorm and eventually build a more robust foundation for sustainable government-academic collaborations. In doing so, we can better prepare for future public health emergencies and ensure that our responses are informed to the point at which they become both efficient and effective.
